# Removal of Methyl Red from Aqueous Solution Using Biochar Derived from Fennel Seeds

**DOI:** 10.3390/molecules28237786

**Published:** 2023-11-26

**Authors:** Dorota Paluch, Aleksandra Bazan-Wozniak, Robert Wolski, Agnieszka Nosal-Wiercińska, Robert Pietrzak

**Affiliations:** 1 Department of Applied Chemistry, Faculty of Chemistry, Adam Mickiewicz University in Poznań, Uniwersytetu Poznańskiego 8, 61-614 Poznan, Poland; dorota.paluch@amu.edu.pl (D.P.); aleksandra.bazan@amu.edu.pl (A.B.-W.); robert.wolski@amu.edu.pl (R.W.); 2 Department of Analytical Chemistry, Institute of Chemical Sciences, Faculty of Chemistry, Maria Curie-Sklodowska University, Maria Curie-Sklodowska Sq. 3, 20-031 Lublin, Poland; agnieszka.nosal-wiercinska@mail.umcs.pl

**Keywords:** biochar, adsorption, chemical and physical activation, methyl red

## Abstract

In this study, fennel (*Foeniculum vulgare*) seeds were used as a precursor to obtain carbon adsorbents through physical activation with carbon dioxide and chemical activation by impregnating the precursor with sodium carbonate. The physical activation involved the carbonization of the precursor at a temperature of 600 °C for 60 min and activation at a temperature of 800 °C for 30 min with carbon dioxide. Chemical activation included impregnation of the precursor with sodium carbonate at a mass ratio of a precursor to activator of 1:2. The mixture was activated in a nitrogen atmosphere with a flow rate at a temperature of 700 °C for 45 min. The resulting biochar samples were washed with 5% hydrochloric acid and subsequently rinsed with boiling distilled water. The biochar adsorbents were characterized using low-temperature nitrogen adsorption–desorption isotherms, Boehm titration, and pH measurements of their aqueous extracts. The specific surface area of the obtained adsorbents ranged from 89 to 345 m^2^/g. Biochar adsorbents exhibit a predominance of acidic groups over basic groups on their surfaces. The sorption capacities of the obtained samples towards an aqueous solution of methyl red range from 26 to 135 mg/g. Based on adsorption studies, it was found that the adsorption of the dye on the obtained biochar materials follows a pseudo-second-order model. The Freundlich isotherm best describes the studied process, indicating the formation of a multilayer of adsorbate on the adsorbent surface. The efficacy of adsorption in aqueous solutions of methyl red was found to increase with the elevation of the process temperature. Moreover, thermodynamic studies have shown that the adsorption process is spontaneous and endothermic. Consequently, this work provides a description of the physicochemical parameters of two biochars obtained by physical and chemical activation of a little-studied precursor—fennel seeds—and studies on their potential use as adsorbents for contaminants from the aqueous phase.

## 1. Introduction

Textile dyes play a key role in the vibrant world of fashion, adding color and character to our clothing and fabrics. However, beneath the allure of vibrant colors lies a less glamorous reality—the severe environmental and health consequences associated with these dyes. With over 10,000 synthetic dye varieties and an annual production of nearly 800,000 tons, a substantial portion ends up as harmful wastewater [1]. Textile dyes, such as naphthalene, benzidine, and other aromatic amines present in the parent molecule or its intermediate metabolites, have been identified as culprits for toxic effects. These dyes, like many other industrial pollutants, are highly toxic and potentially carcinogenic, contributing to environmental degradation and causing various health problems in animals and humans. The use of textile dyes in the textile industry has led to a number of human, animal, and environmental health problems [2]. In 2010, the United Nations designated water and sanitation as the sixth sustainable development goal (SDG), with the objective of guaranteeing accessibility and the sustainable administration of water and sanitation for everyone [3].

The most optimal method of removing contaminants from the aqueous phase should guarantee the efficient elimination of considerable quantities of dye from wastewater within a short period without generating consequential pollutants [4]. The existing techniques for wastewater treatment can be classified as biological, chemical, or physical. Typically, physical methods are the most straightforward and commonly implemented. Several techniques can be applied to remove contaminants from the aqueous phase, such as coagulation, ion exchange, irradiation, membrane filtration, nanofiltration, reverse osmosis, and adsorption [4]. Adsorption is considered the most effective method for removing contaminants from the aqueous phase [5]. Physisorption and chemisorption are the two common types of adsorption, distinguishing how the dye molecules adhere to the adsorbent surface [6]. Various forces, such as hydrophobic interactions, van der Waals forces, electrostatic contacts, and hydrogen bonding, are at play in the adsorption mechanism [6]. The porous structure of most adsorbents allows for efficient dye molecule absorption, as it increases the total open surface area. Silica gel, alumina, zeolites, and activated carbon are commonly used adsorbents for dye removal from textile wastewater [6]. Activated carbon is the most widely used adsorbent, but there are also less expensive alternatives like clay, wood chips, and various agricultural waste materials that can be utilized for dye removal. While using these “relatively inexpensive” adsorbents is cost-effective, it often requires a large volume of adsorbents and may not be as efficient as activated carbon [6].

The production of activated carbon by chemical activation is generally a one-step process that combines carbonization and activation simultaneously. A chemical activating agent, acting as a dehydrating and oxidizing agent, is mixed with the carbon precursor. Chemical activation offers several advantages over physical activation, including a lower activation temperature (<800 °C) in comparison to a physical activation temperature (800–1100 °C) [7]. The textural properties of activated carbon are significantly impacted by its activation conditions. Furthermore, these conditions play a critical role in shaping the carbon’s ultimate properties. The impregnation ratio, activation temperature, and activation time should be taken into account. These optimal conditions vary depending on the specific precursor and activating agent utilized [7].

Due to dwindling fossil carbon resources, alternative precursors to carbon adsorbents, such as activated carbon, biocarbon, and biochar, are now being sought. While numerous scientific papers are annually published on producing carbon adsorbents from plant residues, the preparation of biochar from fennel seeds has not received much attention. This study’s novelty lies in the acquisition of carbon materials (biochar) through the chemical and physical activation of fennel seeds (*Foeniculum vulgare*) via conventional heating methods. Chemical and physical activation were employed to obtain the biochar. Physicochemical examinations were conducted, including BET analysis, scanning electron microscopy (SEM) and Boehm titration. The sorption capacity towards an aqueous solution of methyl red was assessed. The effects of the temperature of the adsorption process and the pH of the aqueous dye solution on the sorption capacity of the obtained adsorbents were determined. In order to determine the mechanism of adsorption of the dye on the obtained biochar samples, characteristic parameters were determined for five isotherm models: Langmuir, Freundlich, Dubinin–Radushkevich, Temkin, and Halsey and Sips. In addition, the kinetics of adsorption of an aqueous solution of methyl red on the obtained biochar adsorbents were determined.

## 2. Results and Discussion

### 2.1. Characterization of the Biochar Samples

Table 1 shows the textural parameters and iodine numbers of the adsorbents obtained by physical (PA) and chemical (CA) activation of fennel seeds. The biochar CA, prepared by impregnating the precursor with sodium carbonate, exhibits superior textural properties compared to the PA sample. The specific surface area of the CA sample measures 345 m^2^/g, whereas the PA sample has a specific surface area of 89 m^2^/g. Additionally, the total pore volume of the CA sample is 2.5 times greater than that of the PA sample. Based on the average pore diameter value, it can be concluded that both materials possess a mesoporous structure. The limited development of the specific surface area in the biochar obtained through the chemical activation of the precursor may be attributed to a low ratio of activator to the precursor. On the other hand, for the sample obtained through the physical activation of the starting material, the relatively small temperature difference between the carbonization and activation processes may account for the observed lower specific surface area.

However, based on the iodine number values shown in Table 1, it can be observed that the biochar obtained through the physical activation of the precursor with carbon dioxide shows an iodine number higher by 94 mg/g than the CA sample obtained through the chemical activation of the precursor with sodium carbonate. It has been reported that the iodine number can be used as an approximation for the surface area of activated carbon through the relationship between 1 m^2^ of surface area per 1 mg of iodine adsorbed [8]. In the case of the CA sample, the correlation between the two parameters is noticeable. However, for the PA biochar, the iodine value obtained significantly exceeds the specific surface area value. The PA sample has pores in its structure that are 64.43% larger compared to the CA sample. Perhaps due to this fact, iodine molecules are more easily incorporated into the structure of the PA adsorbent, resulting in a higher iodine number for this biochar. Moreover, the literature sources suggest that certain functional groups may influence the disproportionately high iodine number compared to the specific surface area [9].

In comparison, the adsorbent obtained by the chemical activation of lignin with sodium carbonate at a precursor:activator weight ratio of 1:2 and using an activation temperature of 800 °C had a specific surface area of 320 m^2^/g [10]. This value is 25 m^2^/g lower than for the CA sample obtained in a similar way but using a lower activation temperature, which is both beneficial from an environmental and economic perspective. Chemical activation of palm oil shell with Na_2_CO_3_, carried out in a manner analogous to that described in the study by Hussaro et al., resulted in a much higher specific surface area of the adsorbent—725 m^2^/g [11]. In this case, the textural properties of the carbon material were influenced by the choice of precursor. Comparing the PA biochar with the adsorbent obtained by the physical activation of biogas plant waste with carbon dioxide at 800 °C analogous to the PA sample, we can see that the adsorbent presented in the paper has an S_BET_ of 320 m^2^/g, which is 231 m^2^/g higher than the PA sample [12].

**Figure 1:**
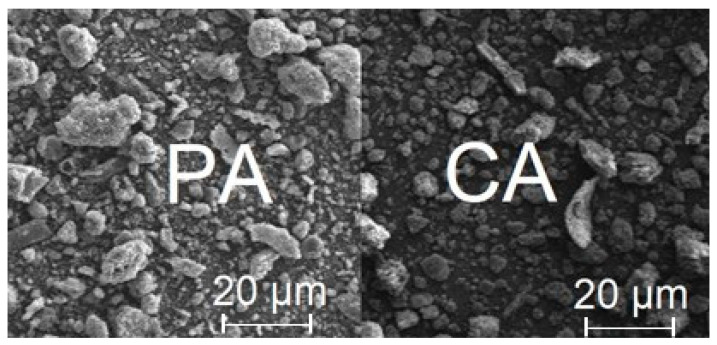
SEM images.

The SEM images of the biochar samples are presented in Figure 1. Regarding the biochar samples, the ash content may account for the brighter fragments observed.

**Figure 2:**
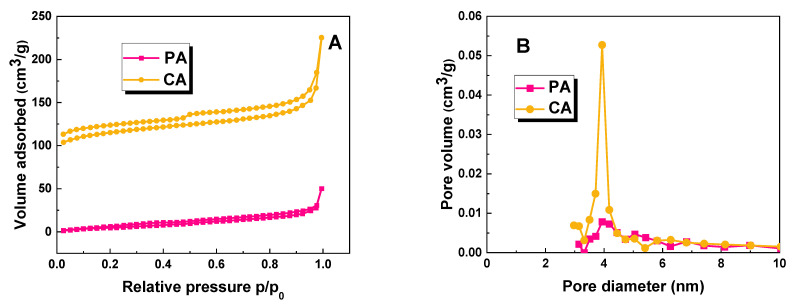
Low-temperature N_2_ adsorption–desorption isotherms (**A**) and pore size distribution (**B**) of biochar samples.

Figure 2 presents the low-temperature nitrogen adsorption/desorption isotherms (A) and the corresponding pore size distribution (B) of the CA and PA samples. According to the nomenclature proposed by Thommes et al. (2015) [13], these isotherms can be classified as type IV(a). The observed hysteresis loop, associated with capillary condensation, indicates the presence of pores larger than 4 nm in diameter. The International Union of Pure and Applied Chemistry (IUPAC) defines six types of hysteresis loops, which are closely linked to the porous structure of the adsorbent and the adsorption mechanism. In this case, all the samples exhibit hysteresis loops of the H4 type, suggesting the presence of narrow gap pores within the biochar materials [13]. The data presented in the graphs strongly support the conclusion that the obtained materials possess a mesoporous structure.

**Figure 3:**
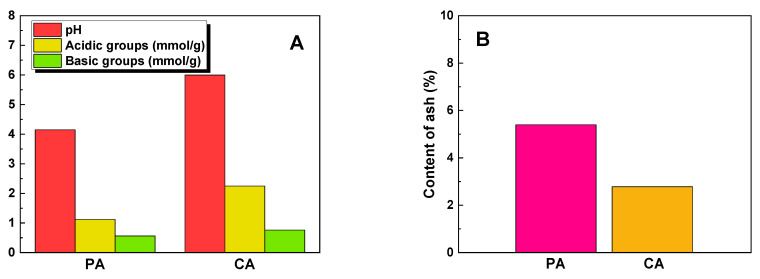
pH values of the aqueous extracts and number of oxygen functional groups on the surface of tested biochar samples (**A**) and content of ash (**B**).

The acid-base properties of the precursor and the obtained biochar samples were investigated, as shown in Figure 3A. The starting material used in this study exhibited a slightly acidic surface character, as indicated by a pH value of 6.1. Furthermore, it displayed a higher content of acidic functional groups compared to basic functional groups. The analysis revealed that both biochar materials also exhibited a clear predominance of acidic groups, with the PA sample having 1.12 mmol/g and the CA sample having 2.25 mmol/g, while the basic groups measured 0.56 mmol/g for PA and 0.76 mmol/g for CA. The biochar obtained through the chemical activation of the precursor showed a higher abundance of oxygen functional groups compared to the biochar derived from the physical activation of fennel seeds. Acidic oxygen functional groups are formed through the oxidation of biochar at high temperatures and contribute to the polar and hydrophilic nature of the adsorbent surface. On the other hand, basic oxygen functional groups are generated as a result of oxygen chemisorption on the adsorbent surface at high temperatures and in an oxidizing gas atmosphere [14].

Ash is an impurity within the biochar structure that has a negative effect on the sorption capacity of the adsorbent [15]. According to the data in Figure 3B, the biochar produced by physical activation of the precursor has a 5.4% ash that is close to that of the precursor (5.9%). However, the chemical activation of the starting material with sodium carbonate leads to a material with an ash content that is nearly half that of the PA sample. 

To investigate the qualitative ash composition of the biochar samples and the precursor, fennel seeds, an ICP analysis was conducted, the results of which are shown in Table 2. As fennel seed is a plant-origin material, the ICP analysis of the precursor’s ash revealed the existence of both macro and micronutrients. Similar conclusions were reported by Azahrani et al. [16]. Upon analysis of Table 2, it is evident that the ash acquired from the precursor contains considerably higher levels of elements in comparison to the biochar obtained. The ash from the precursor is characterized by containing large amounts of potassium, calcium, and phosphorus, while the contents of the other elements are much lower. The mineral substance obtained from the precursor also showed low contents of heavy metals, such as Cu, Cd, Cr, and Pb. In the case of the ash obtained from the biochar samples, the higher elemental content was shown by the PA adsorbent. These values are two/three times higher than for the CA adsorbent. When analyzing the content of heavy metals in both biochar, their content is at very similar levels: in the case of copper, lead, and nickel, an increase in their content in biochar relative to the precursor is noticeable, suggesting their accumulation. This is related to the firing of organic material during biochar production. The organic matter is reduced, which was noted in the determination of the yield of the biochar preparation processes relative to the precursor. The variant of activation used is responsible for the difference in elemental content between the PA and CA samples. The lower elemental content in the CA sample is attributed to the use of chemical activation, while a higher elemental content was detected when carbon dioxide was employed to obtain the PA sample. The difficulty in removing carbonates stems mainly from their insolubility in water. This is especially evident with heavy metals, as their content in the ashes remains practically unchanged. Regarding the materials examined, the silica content remains stable at roughly 70%.

### 2.2. Adsorption Studies

**Figure 4:**
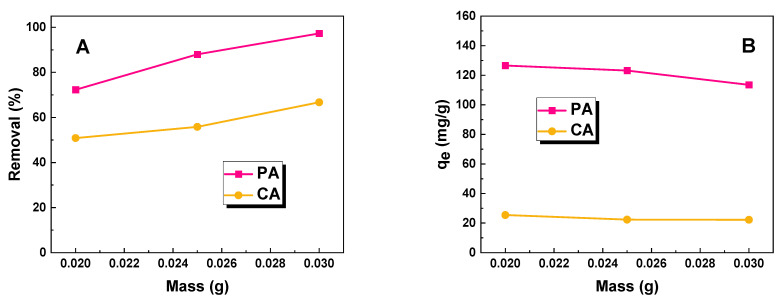
Effect of adsorbent mass (**A**,**B**) on adsorption of methyl red (volume of dye solution: 50 dm^3^; dye concentration for sample PA: 70 mg/dm^3^; dye concentration for sample CA: 20 mg/dm^3^).

Figure 4A,B illustrates the impact of biochar mass on its sorption capacity against an aqueous solution of methyl red at a constant concentration. From the data presented in the graphs, it is evident that for both adsorbents, the system with a biochar mass of 30 mg exhibits the highest effectiveness.

This can be attributed to the increased availability of active sites, resulting from the larger surface area of the adsorbent (Figure 4A). When the mass of the adsorbent is increased at a constant dye concentration, more adsorption sites become available for the adsorbate, leading to an enhanced removal rate [17]. However, it is important to note that the amount of dye per unit mass decreases, as depicted in Figure 4B, resulting in a decrease in the sorption capacity of the biochar samples with an increasing sample mass. Therefore, for optimal results, an adsorbent mass of 25 mg was selected for further studies.

**Figure 5:**
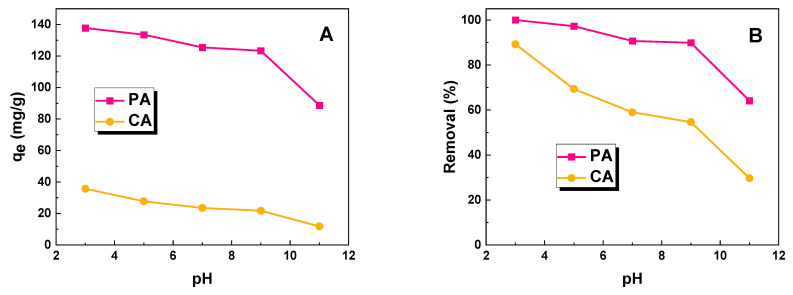
Effect of pH of aqueous dye solution on (**A**) sorption capacities of biochar samples and (**B**) removal of methyl red (volume of dye solution: 50 dm^3^; dye concentration for sample PA: 70 mg/dm^3^; dye concentration for sample CA: 20 mg/dm^3^).

**Figure 6:**
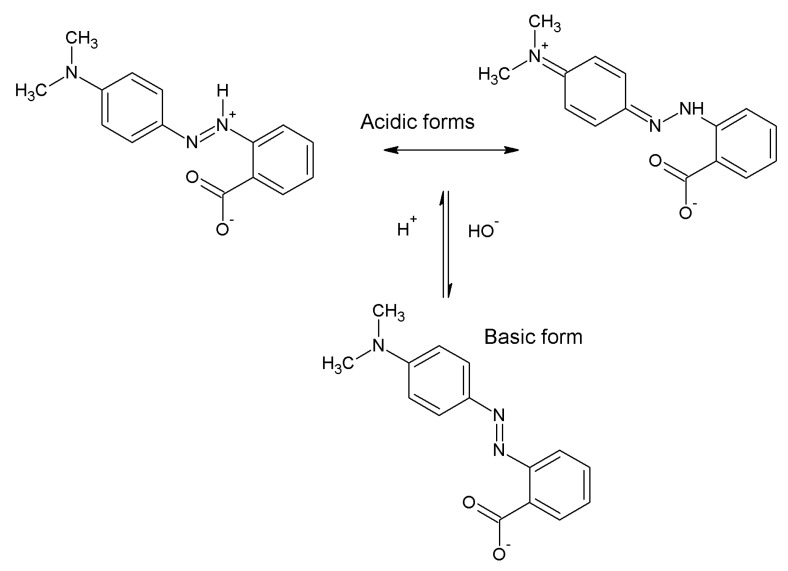
Acid and base forms of methyl red.

In this study, the effect of the pH of the MR solution on its removal efficiency by the obtained biochar was studied. (Figure 5). Based on the results obtained, it can be concluded that for both systems, as the pH of the dye increases, the sorption capacity of the obtained materials decreases. At a low pH, biochar has a positive charge, resulting in more intense adsorption of negatively charged dye molecules, such as methyl red. A similar correlation has been described in other studies [12,18,19,20]. The enhanced adsorption of methyl red on adsorbent surfaces could be attributed to the properties inherent within the molecule. Specifically, when in the presence of protons, the methyl red molecule exhibits both a positive charge on the nitrogen atom and a negative charge in the form of the -COO^−^ group (Figure 6) [21]. As a result of this dual charge structure, the dye molecule is capable of interacting with both acidic and basic oxygen groups on the surface of the biochar.

**Figure 7:**
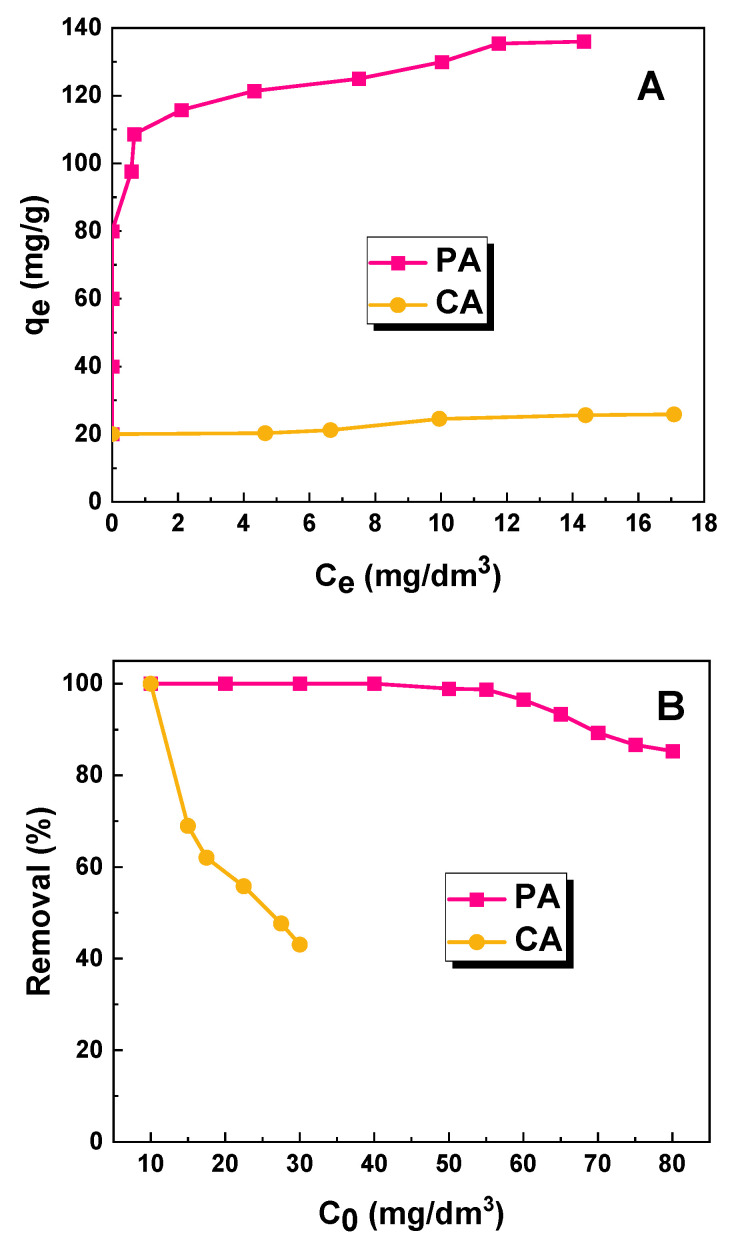
(**A**) Effect of initial concentration on sorption capacities of obtained biochar samples; (**B**) correlation between removal (%) of dye solution and initial concentration.

Figure 7 presents the effect of the initial concentration. The sorption capacity of the biochar obtained through the physical activation of fennel seeds (135 mg/g) is more than five times higher than that of the sample obtained through chemical activation with sodium carbonate (26 mg/g). The high sorption capacity of the PA sample towards the aqueous solution of the dye is a consequence of the acidic nature of the surface of this sample (Figure 3A). The aqueous extract of the PA sample showed a decidedly acidic character, which facilitates the adsorption of anionic dye molecules on the surface of the test material. In contrast, the sorption capacity of the biochar obtained by the chemical activation of fennel seeds with sodium carbonate was 109 mg/g lower than that received for the sample obtained by the physical activation of the precursor. Due to significant differences in the % removal at the concentrations provided (Figure 7B), different reference concentrations were chosen for the subsequent adsorption experiments with each sample. In particular, 70 mg/dm^3^ was chosen as the reference concentration for sample PA, whereas for sample CA, a concentration of 20 mg/dm^3^ was chosen.

This large difference between the sorption capacities of the PA and CA samples is most likely due to the acid-base properties of the materials obtained (Figure 3A), the ash content (Figure 3A), and the pore size (Table 1). Figure 5 indicates an increase in the biochar’s sorption capacity with a decrease in pH value. The pH of the aqueous extract is lower in the PA sample, suggesting a decrease in the solution’s pH during the dye adsorption process that enhances the removal of MR molecules. Additionally, the ash content in the PA sample’s structure is only 5.4%, significantly lower than that observed for the physically activated carbon adsorbents reported in the literature [15]. The low ash content in the material prevented the obstruction of pores in the structure. This, in turn, had a positive impact on its sorption capacity for methyl red, an organic dye. Conversely, the CA sample exhibited low sorption capacity, owing to the presence of groups with a weak acid character on its surface, hindering the efficient capture of dye molecules.

Table 3 shows a comparison of the sorption capacities of the carbon adsorbents obtained from different precursors by means of a chemical activation process using sodium carbonate or a physical activation process using CO_2_. Based on the analysis of the data, it can be concluded that the adsorbent obtained by the chemical activation of fennel seeds with sodium carbonate showed similar dye removal efficiencies compared to the material obtained in an analogous way from caraway seeds [18]. In contrast, the adsorbent obtained by the chemical activation of green tea leaves showed a sorption capacity that is more than double that of the other two adsorbents obtained with the same activator. The higher sorption capacity of the adsorbent described in the paper [18] may be related to the use of a longer activation time for this adsorbent. Comparing the biochar obtained by the physical activation of fennel seeds with the adsorbent obtained in an analogous way from biogas plant waste, it can be concluded that despite the shorter activation time, the PA sample shows a higher sorption capacity of more than 100 mg/g. A study by Bazan-Wozniak et al. showed that the physical activation of residues from the supercritical extraction of marigold at 800 °C for 60 min yields an adsorbent with a sorption capacity of 102 mg/g [22]. A PA sample activated at the same temperature but with a shorter activation time shows a sorption capacity of more than 30 mg/g higher, which is advantageous from an economic point of view, as less energy is needed to achieve better results.

The adsorption of the dye on the biochar samples obtained through physical and chemical activation of the precursor was evaluated by comparing the experimental data with the calculated parameters using the linear forms of the Langmuir, Freundlich, Dubinin–Radushkevich, Temkin, and Halsey and Sips models (Table 4). Based on the data presented, it can be concluded that the adsorption of the aqueous solution of methyl red on the obtained adsorbents follows the Freundlich isotherm model. This is supported by the higher values of the correlation coefficient R^2^ obtained for this model, indicating the formation of an adsorption multilayer on the surface of the biochar. The Freundlich model assumes a homogeneous surface and does not account for the variations in adsorption energy across the adsorbent surface. Moreover, a better fit of the Freundlich model to experimental data suggests that the adsorption process is reversible [23]. The value of 1/*n* in the Freundlich model represents the heterogeneity of the system, with a lower value indicating a greater degree of heterogeneity [24]. The K_F_ constant, determined for the Freundlich isotherm, reflects the strength of the interactions between the adsorbent and adsorbate [25]. A higher value of this parameter indicates a higher adsorption capacity of the adsorbent and, thus, easier removal of the chemical from the system. In the Langmuir isotherm equation, the K_L_ constant represents the maximum amount of a substance that can be bound to a unit area of the adsorbent at equilibrium. A higher value of the K_L_ constant indicates stronger adsorption [26]. Based on the data presented (Table 4), it can be concluded that the adsorption of the dye on the surface of the biochar is more efficient for the PA adsorbent. On the other hand, the system is more homogeneous in the case of the CA sample. The three times higher value of the K_F_ constant for the PA biochar suggests that this sample exhibits more efficient interactions with the adsorbate molecule. In the Temkin model, the B_T_ parameter describes the strength of the interactions between the adsorbate and the adsorbent surface. The higher the value of this parameter, the higher the adsorption energy [24]. The Halsey adsorption isotherm assesses the multilayer adsorption system and delineates its condensation occurring at a considerable distance from the surface. Much like the Freundlich isotherm model, the Halsey model is applicable to scenarios involving multilayer adsorption and heterogeneous surfaces, where the distribution of adsorption heat is non-uniform [27]. For the Dubinin–Radushkevich and Sips models, significantly lower values of the R^2^ parameter were obtained compared to the results obtained for the other isotherms. Therefore, it is presumed that this model does not play a significant role in understanding the adsorption process.

**Figure 8:**
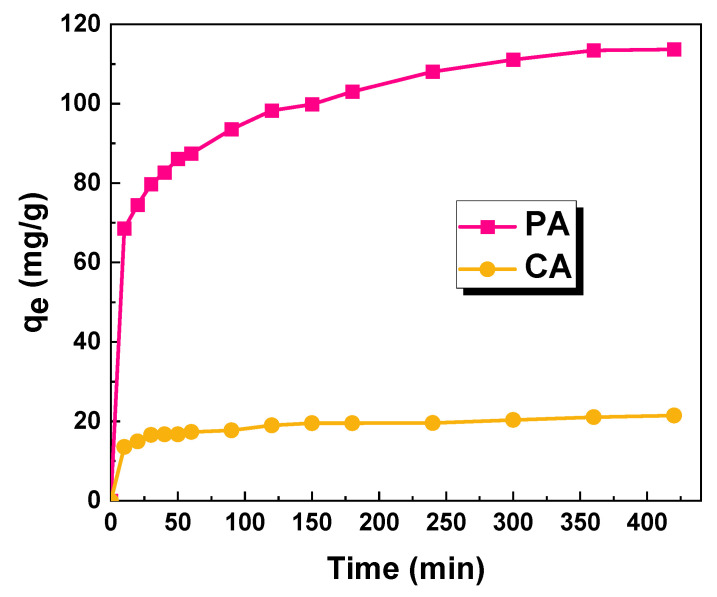
Effect of contact time on the sorption capacities of the biochar samples (biochar mass: 25 mg; volume of dye solution: 50 dm^3^; dye concentration for sample PA: 70 mg/dm^3^; dye concentration for sample CA: 20 mg/dm^3^).

The effect of the adsorbent–adsorbate contact time on the sorption capacities of the obtained biochar samples against methyl red was investigated (Figure 8). Based on the presented results, it can be concluded that, in the case of the CA sample obtained by the chemical activation of fennel seeds with sodium carbonate, the equilibrium of the adsorption process is already established after about 60 min, while in the case of the sample obtained by the physical activation of the starting material, the equilibrium state is established only after about 6 h.

**Figure 9:**
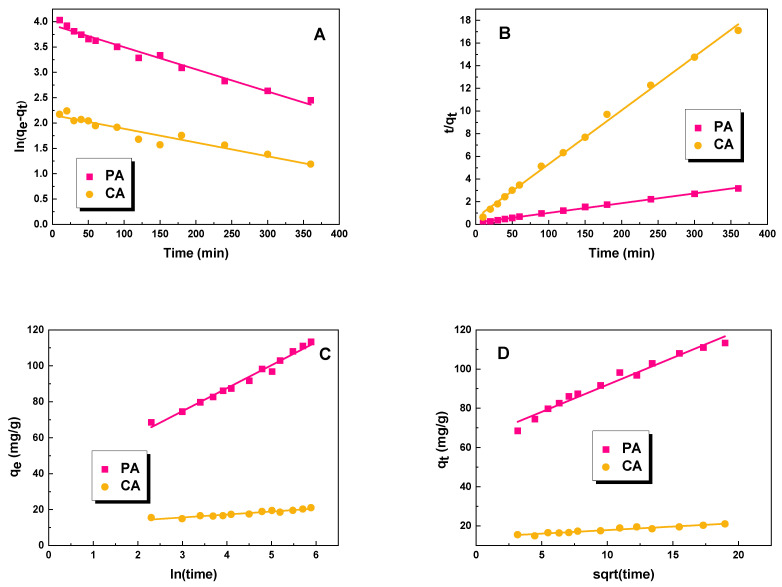
Linear fitting for methyl red on obtained biochar samples to pseudo-first-order (**A**), pseudo-second-order (**B**), Elovich (**C**), and intraparticle diffusion model (**D**).

The resulting graphs for the pseudo-first-order, pseudo-second-order, Elovich, and intraparticle-diffusion linear kinetic models are shown in Figure 9. The constants for the kinetic models are shown in Table 5. Analyzing the data obtained, it can be seen that the values of the correlation coefficient R^2^ for the Elovich model ranged from 0.985 to 0.909, and for intraparticle diffusion, from 0.933 to 0.973, while for the pseudo-first-order model, they ranged from 0.925 to 0.979. These values are significantly lower than the value of the R^2^ correlation coefficient for the pseudo-second-order model, which, for both studied biochar samples, was 0.997. Therefore, we can assume that the process of the adsorption of dye molecules on the obtained adsorbents occurs according to the pseudo-second-order model. This is also confirmed by the theoretically calculated sorption capacity (q_e/cal_), which was close to the experimental values. The pseudo-second-order kinetics constant is an indicator that describes the rate of a chemical reaction. The higher the value of this parameter, the faster the reaction between adsorbent and adsorbate. Based on the results summarized in Table 5, we can conclude that the adsorption of dye on the biochar PA occurs much faster than in the case of sample CA.

However, the correlation coefficient R^2^ is high for both the Elovich and intraparticle diffusion models. The obtained values of *α* (initial adsorption rate) and *β* (desorption constant) from the Elovich model constant indicate that the adsorption process is slower on the surface of PA biochar than on the surface of CA biochar. Additionally, the boundary layer effect is dictated by the values of *C* in the intraparticle diffusion model—higher values lead to a greater effect [28]. Based on the data shown in Table 5, it can be concluded that the PA sample displays a significantly stronger effect. The rate of the diffusion process is determined by the kid constant, which is higher for biochar PA. This information clarifies why biochar PA exhibits a superior sorption capacity to biochar CA.

**Figure 10:**
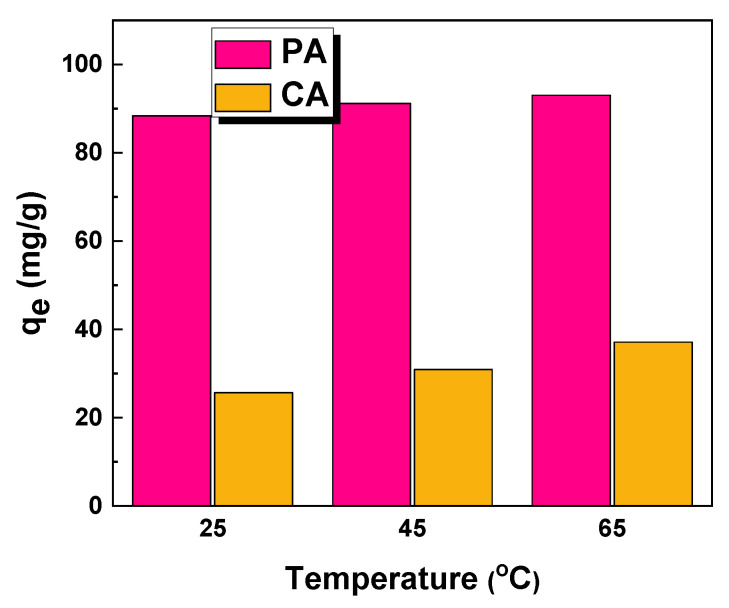
Effects of temperature on adsorption of methyl red (adsorbent mass: 25 mg; volume of dye solution: 50 dm^3^; dye concentration for sample PA: 70 mg/dm^3^; dye concentration for sample CA: 20 mg/dm^3^).

Thermodynamics of the process is another key aspect that provides information on whether the interaction between the guest and host occurs through physisorption or chemisorption. To determine the thermodynamic parameters of adsorption, the process was conducted at three different temperatures: 25, 45, and 65 °C. According to the results (Figure 10), the sorption capacity of the PA biochar for the organic dye showed no significant increase (5.3%). This is desirable from an economic standpoint, as the process is efficient at room temperature and does not require additional energy input. On the other hand, the sorption capacity of the CA adsorbent towards the aqueous dye solution increased by 40%.

The data presented in Table 6 indicate that for both samples, the adsorption process is thermodynamically favorable, as demonstrated by the negative values of the standard free energy (∆*G*^0^), which decrease with increasing process temperature. The increased efficiency of capturing MR molecules from the aqueous solution at higher temperatures suggests an endothermic adsorption process, as supported by the positive values of the enthalpy of adsorption (∆*H*^0^) [29]. When ∆*H*^0^ < 80 kJ/mol, physisorption takes place, while when 80 > ∆*H*^0^ < 200 kJ/mol occurs, chemist precipitation occurs [30]. On this basis, it can be inferred that a process of physisorption occurs on the surface of both biochars. With increasing temperature, the diffusion rate of the MR molecules is enhanced, leading to higher sorption capacities of the resulting samples. The entropy change (∆*S*^0^) is a measure of the system’s disorder and also influences the adsorption process, as MR molecules are attracted and bound to the adsorbent surface [29]. The ∆*S*^0^ value, which measures the change in entropy, was 1.5 times greater for the PA sample. Overall, the thermodynamic analysis indicates that the adsorption of MR onto the biochar samples occurred spontaneously and was endothermic, resulting in greater disorder within the system for the PA sample.

### 2.3. Mechanism of Adsorption

**Figure 11:**
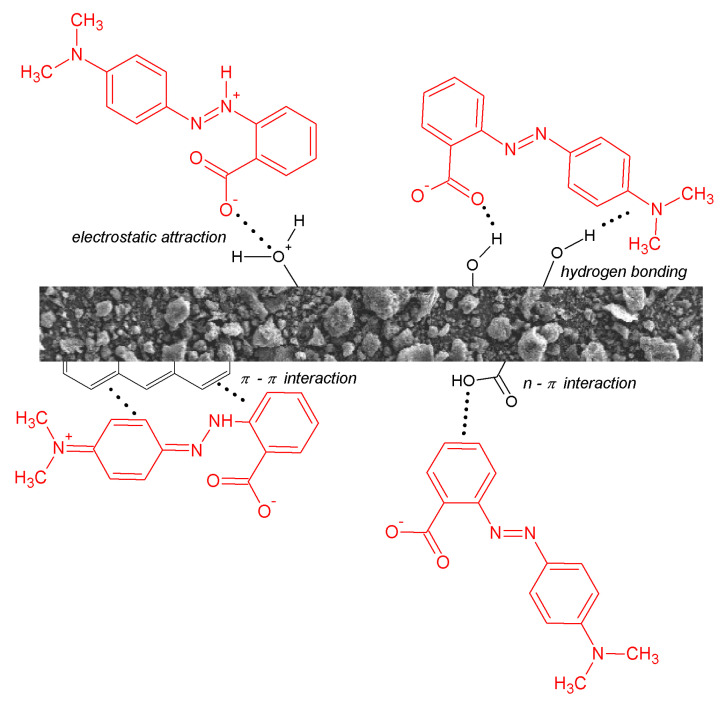
Examples of mechanisms of methyl red adsorption on the surface of biochar.

Figure 11 illustrates the possible interactions between methyl red molecules and the surface of biochar. The mechanism of MR adsorption may involve the electrostatic interaction between the adsorbed dye molecule and the adsorbent surface. The adsorbent surface’s net charge is influenced by its isoelectric point, and it plays a vital role in determining the interaction’s nature [31]. The dye may be adsorbed via various mechanisms: (i) interactions with a positively or negatively charged surface of the adsorbent through electrostatic attraction, (ii) π–π interactions involving aromatic rings found in both the adsorbent and the adsorbate, (iii) hydrogen bonds, or (iv) interactions between an aromatic ring and a heteroatom that contains free electron pairs.

## 3. Materials and Methods

### 3.1. Precursor and Biochar Samples Preparation

The precursor employed in the production of biochar was fennel seeds obtained from Polish crops, which did not meet the quality control standards and, consequently, were considered waste from the herbal industry. The initial characterization of the fennel seeds revealed a volatile matter content of 7.9 wt. %, a moisture content of 5.3 wt. %, and an ash content of 5.8 wt. %. The precursor material was subjected to a 24 h drying period and then divided into two parts for further processing. The first part underwent physical activation (PA). The initial step involved carbonization of the precursor at a temperature of 600 °C with a temperature rate of 10 °C/min in a nitrogen atmosphere with a flow rate of 170 cm^3^/min. The duration of the carbonization process was 60 min. Subsequently, the carbonized material was activated using carbon dioxide at a flow rate of 250 cm^3^/min and a temperature of 800 °C for 30 min with a temperature rate of 10 °C/min. The second part of the precursor underwent chemical activation (CA). Dry fennel seeds were mixed with sodium carbonate at a mass ratio of precursor to activator of 1:2. Water was slowly added to the mixture and heated while being stirred until the reactant fully dissolved. The mixture was subsequently dried and activated in a nitrogen atmosphere with a flow rate of 330 cm^3^/min at a temperature of 700 °C for 45 min with a temperature rate of 10 °C/min. Each experiment was conducted by using a horizontal furnace fitted with quartz, where 15 g of material was placed in a nickel boat. The resulting biochar samples were washed with 5% hydrochloric acid and subsequently rinsed with boiling distilled water. Finally, the materials were dried until a solid mass was obtained, then sieved through a 0.09 mm sieve and homogenized. PA biochar was obtained with a yield of 49.11%, while the CA sample was 32.28%. The adsorption properties of the biochar samples were evaluated using an aqueous solution of methyl red sodium salt (MR), which served as a representative of organic pollutants. The analytical grade methyl red sodium salt was obtained from Merck (Darmstadt, Germany). The rest of the chemicals employed were acquired from Sigma-Aldrich (Burlington, MA, USA), presenting analytical grades.

### 3.2. Characterization of Resulted Biochar Samples

The textural properties of the obtained samples were assessed through nitrogen adsorption/desorption isotherm measurements, conducted at a temperature of −196 °C using an AutosorbiQ analyzer from Quantachrome Instruments (Boynton Beach, FL, USA). Before the analysis, the biochar samples were subjected to vacuum degassing at 300 °C for 12 h. The surface area of the bioadsorbents was determined using the Brunauer–Emmett– Teller (BET) method based on the nitrogen adsorption isotherm. The total pore volume (V_T_) was obtained from the volume of nitrogen adsorbed at a relative pressure of p/p_0_ = 0.99, which is calculated by dividing the equilibrium pressure by the saturation pressure and represents the volume of adsorbed liquid nitrogen at a specific temperature. The average pore diameter (D) was calculated using the equation D = 4V_t_/S_BET_, where S_BET_ represents the surface area of the biochar samples. The pores were assumed to have a cylindrical shape.

SEM images were obtained using a scanning electron microscope (PHILIPS, Eindhoven, The Netherlands) in the following conditions: working distance of 14 mm, acceleration voltage of 15 kV, and digital image recording by DISS.

Total concentrations of the 16 ash elements were determined by high-resolution optical emission spectrometry with inductively coupled plasma (ICP hrOES). A PlasmaQuant PQ 9000 Elite spectrometer (Analytic Jena, Jena, Germany) was used. The following standard conditions were applied: radiofrequency (RF) power of 1.2 kW, plasma gas flow of 12 L/min, auxiliary gas flow of 0.5 L/min, nebulizer gas flow of 0.7 L/min (OneNeb, Agilent, Santa Clara, CA, USA), and an axial view of the plasma. The high-resolution optics consisted of a dual echelle monochromator and a charge-coupled device (CCD), which was cooled to −10 °C using a Peltier system. The following emission lines were used: Al 394.401 nm, Ca 239.856 nm, Cd 214.441 nm, Cr 267.716 nm, Cu 324.754 nm, Fe 262.167 nm, K 769.897 nm, Mg 279.078 nm, Mn 403.075 nm, Na 330.237 nm, Ni 231.604 nm, P 213.618 nm, Pb 220.353 nm, S 182.565 nm, Si 251.611 nm, and Zn 206.200 nm. The signal was measured in 3 repetitions with a measurement time of 2 s. Signal attenuation in the axial view of the plasma was applied for selected elements (Al, Fe, K, and Mg). Yttrium (Y 371.030 nm) was used as the internal standard. The limits of detection (DL) were estimated in a range from 0.007 to 5.6 mg/kg dry weight (DW) using 3-sigma criteria: Al 0.14, Ca 1.4, Cd 0.012, Cr 0.019, Cu 0.019, Fe 0.18, K 4.3, Mg 0.12, Mn, 0.089, Na 5.7, Ni 0.007, P 1.0, Pb 0.26, S 5.6, Si 0.28, and Zn 0.028 mg/kg.

To determine the content of the surface oxygen functional groups with basic and acidic properties in the precursor and biochar materials, the Boehm method was employed. A balance of 0.025 g of the precursor or biochar sample was added to 25 cm^3^ of a 0.1 mol/dm^3^ sodium hydroxide or hydrochloric acid solution, depending on the nature of the functional groups being analyzed. The flasks containing the samples and solutions were tightly sealed and agitated for a period of 24 h. Afterward, the solutions were filtered, and 10 cm^3^ of each filtrate was accurately measured. The excess base or acid present in the solutions was then titrated with a 0.1 mol/dm^3^ hydrochloric acid or sodium hydroxide solution, respectively.

To measure the pH value of the aqueous extracts from the precursor and biochar samples, the following procedure was conducted: 0.1 g of the precursor or biochar material was accurately weighed and added to 10 cm^3^ of distilled water. The resulting suspension was stirred continuously for 24 h to ensure equilibrium. Subsequently, the pH of the aqueous extracts was measured using a pH meter, specifically the Elmetron CP-401 model.

The iodine number was determined according to the ASTM D4607-94 method [15].

### 3.3. Adsorption Studies

The dye selected for testing was methyl red. Aliquots of 25 mg of each biochar sample were added to 50 cm^3^ of an aqueous dye solution with initial concentrations ranging from 10 to 80 mg/dm^3^. The mixtures were then shaken at room temperature (22 ± 1 °C) using a Heidolph laboratory shaker at a speed of 300 rpm/min for a duration of 24 h. Following this, approximately 5 cm^3^ of the solution from each system was collected and centrifuged using a laboratory centrifuge. The absorbance of the supernatant was spectrophotometrically measured at a wavelength of 430 nm using a dual-beam UV-VIS spectrophotometer, specifically the Carry Bio 100 model (Varian). The adsorption capacities (1) and % of the removal (2) of the biochar samples were calculated using the following formulas:

(1) $$q_{e}=\frac{C_{0}-C_{e})}{m}\times V$$

(2) $$\%\;of\;removal=\frac{(C_{0}-C_{e})\times 100\%}{C_{0}}$$

where *C*_0_ and *C_e_* are the initial and equilibrium concentrations (mg/dm^3^) of the dye in the solution, respectively; *m* is the mass of the biochar (g); and *V* is the volume of the solution (dm^3^).

The impact of the pH values (pH values 3–11) of the aqueous solutions of methyl red on the sorption capacity of the produced biochar was ascertained. The measurements were conducted for 25 mg samples of biochar. Each sample was immersed in 50 cm^3^ of MR aqueous solution with a predetermined pH value. The pH of the dye solution was determined by adding 0.1 M HCl or 0.1 M NaOH. The pH value was monitored using the BlueLine 25 pH electrode (SI Analytics, Weilheim, Germany). To determine the sorption capacities of the adsorbents tested against the methyl red solution, five calibration curves were performed—one for each of the pH values tested.

The linear forms of the Langmuir, Freundlich, Dubinin–Radushkevich, Temkin, and Halsey equations were employed to determine a suitable model for the adsorption of dye on biochar. The Langmuir equation assumes that the adsorbent surface possesses a finite number of energetically uniform active sites capable of adsorbing a single molecule of the adsorbate [32]. The Langmuir isotherm can be represented by the following linear Equation (3):

(3) $$\frac{1}{q_{e}}=\frac{1}{q_{max}}+\frac{1}{K_{L}q_{m}}\times \frac{1}{C_{e}}$$

where *q_e_* is the equilibrium amount of adsorbed dye (mg/g), *K_L_* is the Langmuir equilibrium constant (dm^3^/mg), and *q_max_* is the maximum adsorption capacity of the adsorbent (mg/g).

According to the Freundlich model, the number of adsorbed molecules cannot exceed the number of active sites, and the layer formed on the adsorbent surface allows for the formation of additional layers [33]. This model is described by the Equation (4):

(4) $${logq}_{e}={logK}_{F}+\frac{1}{n}{logC}_{e}$$

where *K_F_* is the Freundlich equilibrium constant (mg/g(dm^3^/mg)^1/*n*^), and 1/*n* is the adsorption intensity constant.

The Dubinin–Radushkevich isotherm is derived from the statistical thermodynamics of adsorption and is based on the assumption that adsorption occurs through a series of independent and equivalent sites on the adsorbent surface. It is suitable for modeling adsorption on heterogeneous surfaces with varying adsorption energies [34]. This model is described by the Equation (5):

(5) $${ln\;q}_{e}={ln\;q}_{s}-(\beta \varepsilon^{2})$$

where *β* is a constant describing the sorption energy (mol^2^/kJ^2^), and *ε* is the adsorption potential.

Temkin’s isotherm assumes that the heat of the adsorption for all molecules in the adsorption layer due to the adsorbent–adsorbate interactions decreases linearly, and adsorption is characterized by a uniform distribution of binding energy [35]. This model is described by the Equation (6):

(6) $$q_{e}=B_{T}ln\;A_{T}+B_{T}ln\;Ce$$

where *B_T_* is the Temkin constant (J/mol), and *A_T_* is the Temkin isotherm equilibrium binding constant (L/mg).

The Halsey adsorption isotherm assesses the multilayer adsorption system and characterizes its condensation occurring at a relatively significant distance from the surface [24]. This model is described by the following Equation (7):

(7) $${ln\;q}_{e}={\frac{1}{n_{H}}ln\;K}_{H}-\frac{1}{n_{H}}ln\;Ce$$

where *K_H_* and *n_H_* are Halsey constants.

The Sips is a combined form of the Langmuir and Freundlich model equation [36]. This model is suitable for predicting heterogeneous adsorption systems and localized adsorption without the adsorbate–adsorbate interactions [24]. The Sips isotherm model is given by the following Equation (8):

(8) $$\beta_{s}ln(C_{e})=-ln\;(\frac{K_{s}}{q_{e}})+ln(a_{s})$$

where *q_e_* is the maximum adsorption capacity (mg/g), *K_s_* is the Sips isotherm constant (L/g), *a_s_* is the Sips isotherm exponent (L/g), and *β_s_* is the exponent, which lies between 1 and 0.

In order to characterize the kinetics of methyl red adsorption on the biochar samples, two models were employed: the pseudo-first-order model (9), pseudo-second-order model (10) [37], Elovich model (11) [38], and intraparticle diffusion (12) [39]:

(9) $$\log\left(q_{e}-q_{t}\right)=logq_{e}-\frac{k_{1}}{2.303}t$$

(10) $$\frac{t}{q_{t}}=\frac{1}{k_{2}q_{e^{2}}}+\frac{t}{q_{e}}$$

(11) $$q_{t}=\frac{1}{\beta }ln\;(1+\alpha \beta t)$$

(12) $$q_{t}=k_{id}t^{1/2}+C$$

where *q_e_* is the equilibrium amount of adsorbed dye (mg/g); *q_t_* is the amount of adsorbed dye over time (mg/g); *t* is the process time (min); *k*_1_ is the pseudo-first-order adsorption constant (1/min); *k*_2_ is the pseudo-second-order adsorption constant (g/mg × min); *α* is the Elovich initial sorption rate constant (mg/g × min); *β* is the Elovich desorption constant (g/mg); *k_id_* is the intraparticle diffusion constant (mg/g × min^1/2^); and *C* is the intraparticle diffusion model’s boundary layer constant (mg/g).

Furthermore, the study investigated the impact of sample mass and process temperature on the adsorption of an aqueous solution containing methyl red. In the initial experiment, test samples weighing 20, 25, and 30 mg of each biochar were prepared and immersed in 50 cm3 of the aqueous solution, with concentrations of 70 mg/g for the PA sample and 20 mg/g for the CA sample. The prepared systems were agitated at room temperature (25 °C) using a shaker operating at 300 rpm/min for a duration of 24 h. After this time, about 5 cm^3^ of the solution was taken from each system, centrifuged in a laboratory centrifuge, and then its absorbance was examined spectrophotometrically. In order to determine the effect of the temperature of the aqueous dye solution on the adsorption efficiency, a series of three samples (25 mg) of each biochar were prepared and then immersed analogously to the weighing test. The samples were shaken for 24 h at 25, 45, and 65 °C. Subsequently, the samples were centrifuged in a laboratory centrifuge, and spectrophotometric measurements were conducted.

The thermodynamic parameters were determined using the following formulas for the calculations (13)–(15):

(13) $$∆G^{0}=-RTlnK_{d}$$

(14) $$∆G^{0}=∆H^{0}-T∆S^{0}$$

(15) $$lnK_{d}=\frac{∆S^{0}}{R}+\frac{∆H^{0}}{RT}$$

where Δ*G*^0^ is Gibbs free energy; *R* is the universal constant (8.314 J/mol × K); *T* is the temperature (K); Δ*H*^0^ is the enthalpy change; Δ*S*^0^ is the entropy change; and *K_d_* is the thermodynamic equilibrium constant.

## 4. Conclusions

This study showed that the physicochemical and sorptive properties of the obtained biochar samples depend on the method of activation, which affects their textural parameters and sorptive capacity against an aqueous solution of methyl red sodium salt. In the case of chemical activation of the precursor with sodium carbonate, the obtained adsorbent had a specific surface area of 345 m^2^/g and was able to adsorb only 26 mg of the dye per gram of the sample. The adsorbent obtained by the physical activation of fennel seeds (q_e_ = 135 mg/g), which had a specific surface area of 89 m^2^/g, showed better sorption capacity. This study showed that the adsorption efficiency of an aqueous solution of methyl red on the obtained carbon adsorbents increased with the increasing process temperature. The Freundlich model best described the studied adsorption process, which indicates that an adsorption multilayer is formed on the surface of the adsorbent. In turn, the pseudo-second-order model effectively described the dye removal process. The better sorption capacity of the PA biochar can be attributed to its large pores, low ash content, and prominently acidic surface properties that facilitate the adsorption of the anionic dye methyl red. Furthermore, the calculated kinetic parameters confirm that adsorption onto biochar PA is stronger than adsorption onto biochar CA. The results also showed that it is necessary to optimize the process of producing biochar samples, including the selection of appropriate activation parameters to obtain adsorbents with more favorable textural parameters. In the case of chemical activation of the precursor, the precursor:activator mass ratio or process temperature can be increased. For the physical activation of the starting material, the activation temperature or sample annealing time can be increased.

## Figures and Tables

**Table 1:** Textural parameters and iodine numbers of the obtained biochar samples.

Sample	Surface Area ^1^ (m^2^/g)		Pore Volume (cm^3^/g)		Average Pore Size (nm)	Iodine Number (mg/g)
	Total	Micropore	Total	Micropore		
PA	89	29	0.139	0.081	6.27	465
CA	345	302	0.349	0.165	4.04	371

^1^ Error range between 2 and 5%.

**Table 2:** Content of selected elements in ashes made by ICP method. The % by weight in relation to the weight of the ash sample.

Element	Sample		
	Precursor [%]	PA [%]	CA [%]
Na	0.231	0.012	0.005
K	3.154	0.041	0.026
Ca	2.430	0.279	0.063
Mg	0.971	0.289	0.022
Fe	0.192	0.064	0.047
Mn	0.027	0.006	0.002
Al	0.184	0.066	0.030
Cu	0.002	0.003	0.003
Cd	0.001	0.001	0.001
Cr	0.002	0.001	0.001
Pb	0.001	0.002	0.002
Ni	0.001	0.002	0.001
P	2.199	0.234	0.033
S	0.472	0.060	0.022
Si	0.221	0.135	0.041
Zn	0.005	0.012	0.011
SiO_2_	71	69	70

**Table 3:** Comparison of the carbon materials obtained with various reported adsorbents and their sorption capacities for methyl red.

Precursor	Activator	Precursor: Activator Ratio	Activation Time (min)	Activation Temperature (°C)	Sorption Capacity (mg/g)	Source
Fennel seeds	CO_2_	-	30	800	135	This study
	Na_2_CO_3_	1:2	45	700	26	
Caraway seeds	Na_2_CO_3_	1:2	45	700	28	[18]
Green tea leaves	Na_2_CO_3_	1:2	60	700	70	[19]
Biogas Plant Waste	CO_2_	-	45	800	31	[12]
Marigold	CO_2_	-	60	800	102	[22]

**Table 4:** The values of constants determined for the linear Langmuir, Freundlich, Dubinin–Radushkevich, Temkin, and Halsey and Sips for experimental data of methyl red.

Isotherms	Parameters	Sample	
		PA	CA
	*q_exp_* (mg/g)	135	26
Langmuir	*K_L_* (dm^3^/mg)	1.261	0.473
	*R* ^2^	0.887	0.940
	*q_m_* (mg/g)	130	29
Freundlich	*K_F_* (mg/g(dm^3^/mg)^1/*n*^)	45.04	14.97
	*R* ^2^	0.998	0.997
	1/*n*	0.047	0.191
Dubinin–Radushkevich	*E* (kJ/mol)	1119.04	649.46
	*R* ^2^	0.733	0.882
	*q_m_* (mg/g)	133	26
Temkin	*B_T_* (J/mol)	3.43	9.70
	*R* ^2^	0.887	0.971
	*A_T_* (L/mg)	1.00	1.05
Halsey	*K_H_*	104.47	15.36
	*R* ^2^	0.926	0.943
	*n_H_*	10.03	5.76
Sips	*K_S_* (L/g)	998.49	13.03
	*R* ^2^	0.787	0.695
	*a_S_* (L/g)	0.61	4.48
	*β_S_*	0.77	0.29

**Table 5:** Kinetic model parameters.

Kinetics Model	Parameters	Sample	
		PA	CA
	*q_e_* (mg/g)	113	21
Pseudo-first-order	*k*_1_ (1/min)	1.22 × 10^−5^	7.53 × 10^−6^
	*R* ^2^	0.979	0.925
	*q_e/cal_* (mg/g)	51	8
Pseudo-second-order	*k*_2_ (g/mg × min)	24.56 × 10^−3^	3.52 × 10^−3^
	*R* ^2^	0.997	0.997
	*q_e/cal_* (mg/g)	116	21
Elovich	*α* (mg/g × min)	0.078	0.607
	*R* ^2^	0.985	0.909
	*β* (g/mg)	2.17 × 10^−2^	1.09 × 10^−3^
Intraparticle diffusion	*k_id_* (mg/g × min^1/2^)	2.76	0.36
	*R* ^2^	0.973	0.933
	*C* (mg/g)	64.33	14.23

**Table 6:** Thermodynamic parameters of adsorption of aqueous solution of methyl red on the obtained biochar samples.

Sample	Temperature (°C)	∆*G*^0^ (kJ/mol)	∆*H*^0^ (kJ/mol)	∆*S*^0^ (J/mol × K)
PA	25	−8.85	63.06	239.95
	45	−12.33		
	65	−18.56		
CA	25	−3.20	42.88	153.48
	45	−5.13		
	65	−9.44		

## Data Availability

Data are contained within this article.
